# RNA干扰*ERCC1*基因表达对肺腺癌细胞A549/DDP顺铂耐药的影响

**DOI:** 10.3779/j.issn.1009-3419.2010.09.02

**Published:** 2010-09-20

**Authors:** 赟 高, 丹 苏, 莉莎 应, 汪霞 吕, 胜林 马

**Affiliations:** 1 310022 杭州，浙江省肿瘤医院肿瘤研究所 Cancer Research Institute, Zhejiang Cancer Hospital, Hangzhou 310022, China; 2 310022 杭州，浙江省肿瘤医院肿瘤内科 Department of Medical Oncology, Zhejiang Cancer Hospital, Hangzhou 310022, China

**Keywords:** *ERCC1*基因, RNA干扰, 顺铂, 肺肿瘤, *ERCC1* gene, RNAi, Cisplatin, Lung neoplasms

## Abstract

**背景与目的:**

核苷酸切除修复机制可修复顺铂(DDP)造成的DNA损伤，作为其中的关键酶之一，切除修复交叉互补基因1(excision repair cross-complementing gene 1, *ERCC1*)的表达可能与DDP耐药有关。本研究旨在探讨小干扰RNA沉默*ERCC1*基因表达对肺腺癌耐药细胞A549/DDP药物敏感性的影响。

**方法:**

将体外设计合成针对ERCC1的小分子RNA(siRNA)转染A549/DDP细胞，RT-PCR检测ERCC1 mRNA水平；Western blot检测ERCC1蛋白表达的变化；MTT检测RNA干扰后细胞药物敏感性改变。

**结果:**

ERCC1 RNAi干扰后ERCC1 mRNA表达指数降低，明显低于对照组，干扰后ERCC1 mRNA表达抑制率为90.4%。转染ERCC1 siRNA降低了ERCC1蛋白的表达水平。MTT显示分别用2 μg/mL、4 μg/mL、8 μg/mL、16 μg/mL、32 μg/mL顺铂处理细胞48 h，转染ERCC1 siRNA细胞组较对照组敏感性明显增高，干扰前A549/DDP细胞IC_50_为12.49 μg/mL，干扰后A549/DDP细胞IC_50_下降为9.27 μg/mL。

**结论:**

ERCC1 siRNA干扰可以降低ERCC1的表达水平，并可提高A549/DDP细胞对顺铂的敏感性。

## Abstract (en)

**Background and objective:**

The excision repair cross-complementing gene 1 (*ERCC1*), which is important in the repair of cisplatin-DNA adducts, was reported to be related to cisplatin resistance in tumor cells. The aim of this study is to investigate the changes of cisplatin sensitivity by silencing *ERCC1* gene in lung cancer cell.

**Methods:**

The small interfering RNA (siRNA) targeting *ERCC1* gene was designed and synthesized, and transfected to lung cancer cell A549/DDP. The mRNA and protein expression levels of ERCC1 were evaluated by RT-PCR and Western blot. The changes of cisplatin sensitivity after RNA interference were examined by methyl thiazolyl assay.

**Results:**

In A549/DDP cell, the mRNA and protein levels of ERCC1 were decreased and the sensitivity to cisplatin was increased from 12.49 μg/mL to 9.27 μg/mL after transfection.

**Conclusion:**

The sensitivity to cisplatin of lung cancer cell A549/DDP could be enhanced by RNA interfering *ERCC1* gene targeted code 346.

肺癌目前的发病率和死亡率在我国男性中均较高，约65%的肺癌患者确诊时已属晚期，失去了手术机会。化疗在肺癌的综合治疗中占有重要地位，但多药耐药是影响其疗效的主要障碍。越来越多的研究^[1-3]^表明，切除修复交叉互补基因1 (excision repair cross complementing gene 1, *ERCC1*)表达水平与肿瘤顺铂(cisplatin, DDP)耐药以及肺癌患者生存时间相关。RNA干扰(RNA interference, RNAi)技术是近年来较为热门的一种研究基因功能和进行基因治疗的新方法。本研究拟通过RNA干扰技术降低*ERCC1* 基因的表达，探讨干扰前后人肺癌耐顺铂细胞株A549/DDP对顺铂的敏感性变化，为克服肺癌顺铂耐药提供新思路。

## 1 材料与方法

### 1.1 材料

#### 1.1.1 细胞株

人肺癌耐顺铂细胞株A549/DDP由同济大学上海肺科医院周彩存教授惠赠，在含15%小牛血清的RPMI-1640培养基中培养，培养条件为37 ℃、5%CO_2_。

#### 1.1.2 试剂与仪器

siRNA由Ambion公司设计合成；阳离子脂质体转染试剂Lipofectamine 2000、Trizol购于Invitrogen公司；反转录试剂购于Promega公司，荧光PCR试剂购于Bioer公司，其它试剂均为进口分装或国产分析纯。7700荧光定量PCR仪为ABI公司产品；多功能酶标仪为Thermo公司产品。

### 1.2 方法

#### 1.2.1 siRNA的设计、合成

由Ambion公司完成。

#### 1.2.2 转染

采用Lipofectamine 2000脂质体转染法。当A549/DDP细胞融合30%-50%(此时细胞处于对数生长期，状态最佳)在6孔板中进行转染实验。转染步骤按说明书操作。

#### 1.2.3 RT-PCR检测RNAi后A549/DDP细胞*ERCC1*基因和*GAPDH*基因的表达

转染48 h后每组分别收集细胞，提取细胞总RNA，逆转录为cDNA。以GAPDH为内参照扩增*ERCC1*基因。扩增条件为：95 ℃预变性10 min，95 ℃变性15 s，60 ℃退火45 s，72 ℃延伸45 s，40个循环后72 ℃延伸10 min。PCR产物作溶解曲线分析是否为引物二聚体。根据Ct值进行相对定量分析，各组ERCC1 mRNA相对表达量以相对于未转染组的倍数2^-ΔΔCt^表示，ΔCt=Ct_ERCC1_-Ct_GAPDH_，ΔΔCt=ΔCt_RNAi组_-ΔCt_脂质体组_，抑制效率=1-2^-ΔΔCt^。

#### 1.2.4 Western blot检测RNAi前后A549/DDP细胞ERCC1蛋白表达水平变化

收集对数生长期A549和A549/DDP细胞，用1 mL 1×PBS收集下来后在4 ℃、2 000 rpm转速下离心5 min，弃去上清。加入适量的含有蛋白酶抑制剂的细胞裂解液。在冰上用细胞超声粉碎仪进行超声裂解直至细胞液变成澄清透明，4 ℃、13 000 rpm转速下离心30 min。将上清转移至新管，用5×SDS电泳样品缓冲液在沸水中煮沸5 min变性，-20 ℃保存备用。BCA蛋白浓度测定试剂盒检测收集的蛋白浓度，蛋白上样量为30 μg每孔，依次进行电泳及转膜、封闭及抗体孵育、显影。Bio-Rad公司Quantity One软件分析转染组与对照组蛋白表达强度。

#### 1.2.5 MTT法检测RNAi后A549/DDP细胞对顺铂敏感性的变化

取对数生长期的A549/DDP细胞，以4×10^3^/孔接种于96孔板上，24 h后换成无血清1640培养液，48 h后按说明书操作进行转染。转染8 h后加入不同浓度顺铂(终浓度分别为32 μg/mL、16 μg/mL、8 μg/mL、4 μg/mL、2 μg/mL、1 μg/mL)继续培养48 h。每孔加入5 mg/mL四甲基偶氮唑蓝(MTT) 20 μL，4 h后加DMSO震荡10 min，酶标仪测定570 nm处的吸光度，计算半数抑制量(IC_50_)值。

### 1.3 统计学处理

统计使用SPSS 12.0统计软件，数据以Mean±SD表示，统计学分析采用*t*检验，以*P*＜0.05为差异有统计学意义。

## 2 结果

### 2.1 RNAi后A549/DDP细胞*ERCC1*基因的表达变化

根据2^-ΔΔCt^值的大小，得到三组细胞*ERCC1*基因表达的相对差异(图 1)。与脂质体组相比，RNA干扰组细胞ERCC1基因表达降低，其中针对*ERCC1*基因第346核苷酸设计的siRNA(RNAi 1组)干扰效率要高于针对第281核苷酸设计的siRNA(RNAi 2组)。

**1:**
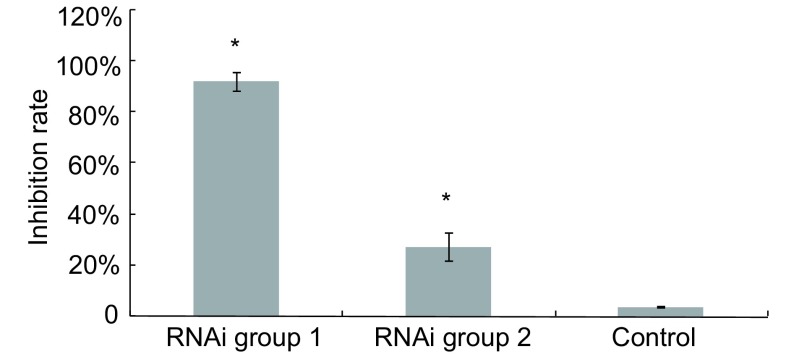
不同siRNA对*ERCC1*基因表达的抑制效率 The inhibition rates of two siRNAs on the expression of *ERCC1* gene. ^*^: *P* < 0.05, *vs* control.

### 2.2 RNAi后A549/DDP细胞ERCC1蛋白表达水平改变

转染前，A549/DDP细胞中ERCC1蛋白相对表达强度为0.710±0.057，转染ERCC1 siRNA后降为0.495±0.102，ERCC1蛋白表达明显降低(*P*＜0.05)(图 2)。

**2:**
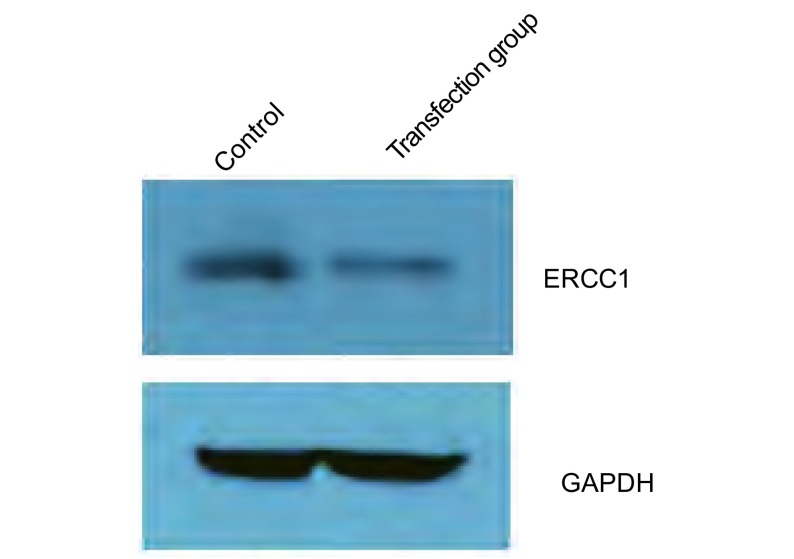
A549/DDP转染ERCC1 siRNA前后ERCC1蛋白表达水平的变化 ERCC1 protein expression levels of A549/DDP before and after transfection with siRNA ERCC1

### 2.3 RNAi后A549/DDP细胞对顺铂敏感性的变化

**1:** 针对靶基因ERCC1 mRNA设计2对siRNA序列 Two pairs of ERCC1 siRNAsequences designed according to target gene ERCC1 mRNA

Group	Targeted nucleotide locus	Percent G/C	siRNA sequences
RNAi group 1	346	48% 53%	Sence: CCUACGCCGAAUAUGCCAU Antisence: AUGGCAUAUUCGGCGUAGG
RNAi group 2	281	43%	Sence: GCCCUUAUUCCGAUCUACA
		43%	Antisence: UGUAGAUCGGAAUAAGGGC

转染ERCC1 siRNA后，RNAi组A549/DDP细胞对顺铂的IC_50_值为9.27 μg/mL，对照组细胞的IC_50_值为12.49 μg/mL，转染后细胞对顺铂的敏感性增加了1.35倍。RNAi组和对照组细胞的抑制率见图 3。

**3:**
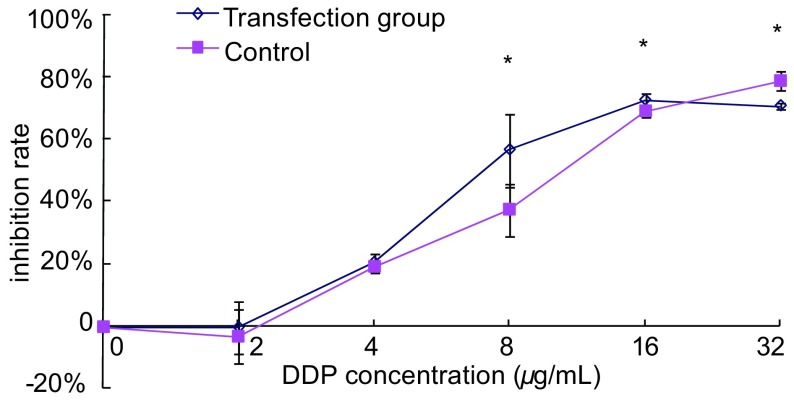
不同浓度顺铂对RNA干扰后A549/DDP细胞的抑制率 The effect of cisplatin on A549/DDP cell proliferation transfected by siRNA. ^*^P < 0.05, *vs* control.

## 3 讨论

化疗在肺癌的治疗中占有重要地位，单药化疗中最有效的是DDP，目前最常以DDP为基础组成联合方案。耐药是影响化疗疗效和肺癌患者生存率的主要原因。目前研究^[4-7]^认为顺铂耐药机制主要包括：药物外排增加、耐药相关基因的改变、DNA损伤修复功能增强、细胞凋亡受抑制等。其中，DNA损伤修复功能是肺癌顺铂耐药机制研究及肺癌个体化治疗的热点。DNA修复主要存在5种途径：核苷酸切除修复(nucleotide excision repair, NER)、错配修复、碱基切除修复、同源NER重组修复和非同源末端连接。其中NER可切除修复各种结构不相关的DNA损伤，特别是在庞大的DNA共价损伤的修复中发挥重要作用，比如紫外线照射诱发的嘧啶二聚体、多环芳香碳氢化合物、氧化损伤以及铂类药物所致的DNA损伤。ERCC1、ERCC2、ERCC3等20多种蛋白参与其中，而DNA损伤的识别/切除是限速步骤，使得在该步骤中起主导作用的ERCC1最为引人瞩目，其功能活性的高低可反映整个NER修复活性的水平^[8]^。

目前，有效抑制基因表达的方法主要有：基因敲除^[9]^、反义核苷酸技术^[10]^和RNAi技术^[11, 12]^。其中，RNAi技术比基因敲除技术易于操作，而且基因抑制效果好于反义核苷酸技术。将与mRNA序列对应的正义和反义RNA组成的双链RNA导入细胞，可以使RNA降解和基因沉默，这种转录后基因沉默(post-transcriptional gene silencing, PTGS)被称为RNAi^[13, 14]^。siRNA的设计是RNAi技术成败的关键。根据siRNA的设计原则，本实验设计合成2对siRNA序列。通过筛选，我们认为针对*ERCC1*基因346核苷酸设计的siRNA能更有效地抑制该基因表达，抑制效率更高。RT-PCR和Western blot结果均证明，转染ERCC1 siRNA后，与对照组相比人肺腺癌细胞株A549/DDP细胞*ERCC1*基因和蛋白表达水平明显降低，表明通过RNAi后*ERCC1*基因的转录和翻译均被抑制。

A549/DDP细胞是用小剂量DDP作为诱导剂、对人肺腺癌细胞A549进行逐步长期诱导而建立的耐DDP人肺腺癌细胞株。MTT实验结果显示在一定的剂量范围内，随着DDP浓度的升高，顺铂对RNAi后A549/DDP细胞的抑制率增加，研究证实降低*ERCC1*基因表达水平可以提高铂类药物化疗敏感性，ERCC1可以作为肺癌个体化治疗时是否选择顺铂的预测指标之一。应用siRNA干扰技术诱导*ERCC1*基因表达下调，可增加肺癌细胞等肿瘤对铂类药物的敏感性，具有一定的临床潜在应用价值，为今后非小细胞肺癌的个体化治疗提供了一定的理论依据。实验发现当DDP浓度达到一定的剂量后抑制率反而降低，我们认为RNAi技术对*ERCC1*基因的不完全抑制及其它耐药机制的存在导致了A549/DDP细胞耐药逆转的不彻底性。另外，肿瘤耐药不是单基因作用的结果，还有其它因素的参与，封闭单个基因不能完全逆转耐药。因此，ERCC1 siRNA可作为治疗肺癌耐药的新策略，但是其逆转耐药的不彻底性仍是需要克服的主要问题，使用多基因多靶点siRNA可能是一种可行的选择。
